# Association between antibiotic consumption and the rate of carbapenem-resistant Gram-negative bacteria from China based on 153 tertiary hospitals data in 2014

**DOI:** 10.1186/s13756-018-0430-1

**Published:** 2018-11-19

**Authors:** Ping Yang, Yunbo Chen, Saiping Jiang, Ping Shen, Xiaoyang Lu, Yonghong Xiao

**Affiliations:** 1 Department of Pharmacy, the First Affiliated Hospital of Medicine School, Zhejiang University, Hangzhou, China; 2 State Key Laboratory for Diagnosis and Treatment of Infectious Diseases, the First Affiliated Hospital of Medicine School, Zhejiang University, 79 Qingchun Road, Hangzhou, China

**Keywords:** Carbapenem-resistance, *Escherichia coli*, *Klebsiella pneumoniae*, *Pseudomonas aeruginosa*, *Acinetobacter baumannii*, Carbapenem antibiotic consumption

## Abstract

**Background:**

This study aimed to investigate the relationship between the rate of carbapenem-resistant Gram-negative bacteria and antibiotic consumption intensity in 153 tertiary hospitals from China in 2014.

**Methods:**

A retrospective study using national surveillance data from 2014 was conducted. Data on the annual consumption of each antibiotic, as well as the rate of carbapenem-resistant Gram-negative bacteria, were collected from each participating hospital, and the correlation between antibiotic consumption and carbapenem- resistant rate was analyzed.

**Results:**

The overall antibiotic consumption intensity among the hospitals varied between 23.93 and 86.80 defined daily dosages (DDDs) per 100 patient-days (median, 46.30 DDDs per 100 patient-days). Cephalosporins were the most commonly used antibiotic, followed by quinolones, penicillins, and carbapenems, and the rate of carbapenem-resistant Gram-negative bacteria from each hospital varied. The correlations between carbapenem consumption intensity and rate of carbapenem resistance revealed correlation factors of 0.271 for *Escherichia coli* (*p* < 0.01), 0.427 for *Klebsiella pneumoniae* (*p* < 0.01), 0.463 for *Pseudomonas aeruginosa* (*p* < 0.01), and 0.331 for *Acinetobacter baumannii* (*p* < 0.01).

**Conclusions:**

A significant relationship existed between the carbapenem consumption and the rates of carbapenem-resistant gram negative bacilli. Rational use of carbapenems should be implemented to address the issue of carbapenem resistance in hospitals.

**Electronic supplementary material:**

The online version of this article (10.1186/s13756-018-0430-1) contains supplementary material, which is available to authorized users.

## Background

Carbapenem is a beta-lactam antibiotic with a broad antimicrobial spectrum and effective antibacterial activity. It is generally administered as a last resort for treating drug-resistant Gram-negative bacterial infections. However, in recent years, the rate of carbapenem-resistant bacteria has steadily increased [1, 2]. Antibiotic resistance greatly limits therapeutic options, consequently resulting in higher patient morbidity, mortality and considerable economic burden [3].

According to a CHINET surveillance in 2016, *Escherichia coli, Klebsiella* spp., *Acinetobacter* spp., and *Pseudomonas aeruginosa* were the top 4 Gram-negative bacterial species found in all clinical samples obtained from Chinese hospitals. These bacterial species are the leading cause of nosocomial infections. The overall prevalence of carbapenem-resistant strains was 1.8% in *E. coli*, 17.9% in *K. pneumoniae,* 28.7% in *P. aeruginosa*, and 70.8% in *A. baumannii* [4]*.* Mortality from carbapenem-resistant Enterobacteriaceae (CRE) infection was reported by the China CRE Network to be as high as 33.5%, and most cases were determined to be caused by carbapenem-resistant *E. coli* (CREC) and *K. pneumoniae* (CRKP) [5]. A meta-analysis demonstrated a > 2-fold increased risk of mortality with multidrug-resistant *P. aeruginosa* infection compared to susceptible *P. aeruginosa* [6]. In yet another study, the mortality of patients with carbapenem-resistant *A. baumannii* (CRAB) infection was as high as 16–76% [7].

The irrational use of antibiotics can increase selective pressure of bacterial resistance, which is one of the important factors responsible for antimicrobial resistance (AMR). Increasing evidence indicates that antimicrobial drug consumption is associated with AMR [8–11]. Unfortunately, most related studies involve a single hospital, and relatively few researchers have sought to analyze findings from multiple hospitals or a region. In addition, whether antibiotic consumption intensity contributes to AMR has not been widely evaluated, especially multicenter researches in China.

To determine the association between antibiotic consumption intensity and AMR, we collected data on antibiotic consumption and noted the resistances of four Gram-negative bacterial species from 153 tertiary hospitals in China. We then evaluated the correlation between rate of carbapenem resistance and antibiotic consumption intensity.

## Methods

### Study design

A total of 153 hospitals voluntarily participated in this cross-sectional study. Antibiotic consumption and the rate of carbapenem resistance of *E. coli*, *K. pneumoniae*, *P. aeruginosa*, and *A. baumannii* from 153 Chinese tertiary hospitals in 2014 were collected. The correlation between antibiotic consumption and resistance rate was then statistically analyzed.

### Data collection

Data on antibiotic consumption were obtained from the national antibacterial drug clinical consumption survey network. Hospitals are required to report bacterial resistance data to the China AMR surveillance system, and those participating in this study were asked to report data for the whole year of 2014.

For each participating hospital, administrative data, including hospital type, administrative region, number of beds, admissions, and patient-days, were recorded.

### Measurement of antibiotic consumption

The pharmacists of each hospital are required to report the annual consumption of each antibiotic to the national antibacterial drug clinical consumption survey network. Antibiotics were categorized according to the Anatomic Therapeutic Chemical (ATC) classification system [12]. Antibiotic consumption was characterized by antibiotic consumption intensity, which is the number of defined daily dosages (DDDs) per 100 patient-days. According to the World Health Organization ATC/DDD classification, DDD is the assumed average maintenance dose per day for a drug used for its main indication in adults. Patient-days were defined as number of discharged patients during the same period multiplied by the average length of stay of hospitalized patients in the same period. Then, data on the main classes of antibiotic consumption were analyzed.

### Antibiotic resistance

Each participating hospital was asked to collect data on four major Gram-negative bacterial species causing infections (i.e., *E. coli, K. pneumoniae, P. aeruginosa*, and *A. baumannii*) from all sample sources (e.g., bloodstream, respiratory tract, urinary tract, wound pus, other sterile body fluids, cerebrospinal fluid, faeces, genital tract and others). The isolates were nonduplicate samples, as several isolates of each species from each patient who recovered within 7 days were considered one isolate. Data included information on the number of carbapenem-resistant isolates and the total number of bacterial strains isolated from clinical specimens. Carbapenem resistance was defined as a strain resistant to imipenem or meropenem. If the resistance rates were different, the higher resistance rate was selected as the resistance rate of the strain to carbapenems. Rate of resistance was calculated as the number of carbapenem-resistant isolates divided by the total number of isolates of the same species tested multiplied by 100. Antibiotic susceptibility tests are performed as a routine laboratory method in each hospital, and all hospitals must adhere to the Clinical and Laboratory Standards Institute 2014 guidelines. The consistency and standardized assessment of the AMR data are ensured through quality control in each laboratory. The antibiotic resistance data were processed using WHONET 5.6 software, and the quality control bacterial strains were *E. coli* ATCC25922*, K. pneumoniae* ATCC700603*, P. aeruginosa* ATCC27853, and *A. baumannii* ATCC19606. Hospital data with less than 50 isolates per year in total were excluded from the study.

### Statistical analysis

Pearson’s correlation analysis was performed to investigate the association between annual antibiotic consumption and rate of carbapenem resistance in 153 hospitals of China. Specifically, the relationship between the rate of carbapenem resistance and the consumption intensity of overall antibiotic, beta-lactam antibiotics, penicillins, cephalosporins, carbapenems, and fluoroquinolones were analyzed individually. Differences with *p* < 0.05 were considered to indicate statistical significance, and all analyses were conducted using Microsoft Excel 2013 and STATA 20.0 (StataCorp LLC, Texas, USA).

## Results

### Participating hospitals

A total of 153 hospitals voluntarily participated in this study; of these hospitals, 149 were first-class tertiary hospitals, which are described as the highest-quality hospitals in China. A total of 29 hospitals were from North China, 29 hospitals were from East China, 22 hospitals were from Central China, 26 hospitals were from Southern China, 14 hospitals were from Southwest China, 16 hospitals were from Northwest China, and 17 hospitals were from Northeast China. These hospitals had a median of 2356 beds (range, 720–8475 beds) and a median of 90,000 inpatients per year (range, 10,000–410,000). As shown in Additional file 1.

### Antibiotic consumption

During the study period, the overall antibiotic consumption intensity of the hospitals varied between 23.93–86.80 DDDs per 100 patient-days (median, 46.30 DDDs per 100 patient-days). Cephalosporins were the most commonly used antibiotics, followed by fluoroquinolones, penicillins, and carbapenems. The antibiotic consumption intensities for the main antibiotic classes are presented in Table 1.

**Table 1:** Antibiotics consumption intensity for the main classes of antibiotics in 153 hospitals

Class (ATC category)	Antibiotics consumption intensity, median value (range, DDDs per 100 patient-days)
All antibiotics(J01)	46.30 (23.93–86.80)
β-Lactams(J01C + J01D)	33.21 (19.35–67.05)
Penicillins(J01C)	5.52 (0.61–16.89)
Cephalosporins(J01DB + J01 DC + J01DD + J01DE)	24.93 (10.81–52.52)
The third generation cephalosporin(J01DD)	11.16 (2.57–38.98)
The fourth generation cephalosporin(J01DE)	0.27 (0–5.98)
Carbapenems(J01DH)	1.96 (0.17–10.06)
Fluoroquinolones(J01MA)	5.71 (1.58–13.90)

### Isolated strains

**Fig. 1:**
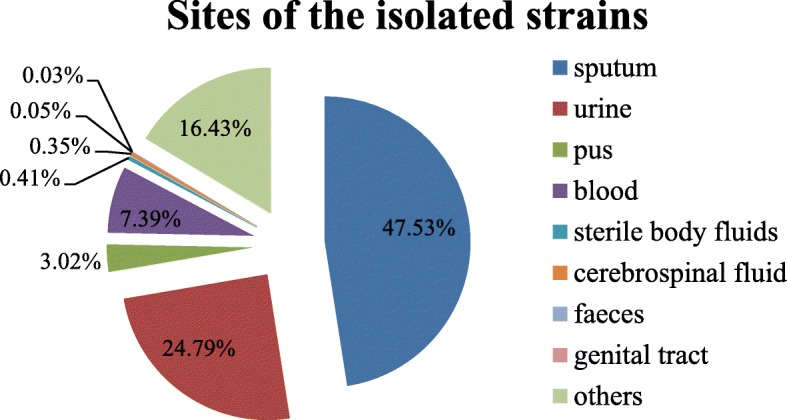
Sites of the isolated strains

A total of 159,199 *E. coli,* 112,135 *K. pneumoniae,*112,792 *P. aeruginosa* and 105,869 *A. baumannii* were analyzed*.* Among the 489,995 isolated strains, the most frequently isolated specimen was sputum (47.53%), then was urine (24.79%), blood (7.39%), pus (3.02%) and so on, as shown in Fig. 1.

### Correlation between antibiotic consumption intensity and carbapenem-resistant gram-negative bacteria

The relationships between the rate of CREC, CRKP, Carbapenem-resistant *P. aeruginosa* (CRPA) and CRAB, and the consumption of various antibiotics are shown in Table 2. As shown in Table 2, carbapenem consumption intensity and the carbapenem-resistant rate of the four Gram-negative bacterial strains positively correlated.

**Table 2:** Correlations between main classes of antibiotics consumption intensity and the rate of carbapenem-resistant in *Escherichia coli, Klebsiella pneumoniae, Pseudomonas aeruginosa* and *Acinetobacter baumannii*

Classes (ATC category)	Correlation							
	*E. coli*		*K. pneumoniae*		*P. aeruginosa*		*A. baumannii*	
	*r* ^a^	p*	*r*	*p*	*r*	*p*	*r*	*p*
All antibiotics (J01)	0.093	0.324	−0.005	0.954	0.061	0.484	0.075	0.416
β-Lactams (J01C + J01D)	0.138	0.141	0.018	0.839	0.021	0.811	0.104	0.260
Penicillins (J01C)	0.121	0.198	−0.041	0.639	−0.044	0.608	−0.104	0.256
Cephalosporins (J01DB + J01 DC + J01DD + J01DE)	0.039	0.676	−0.037	0.678	−0.070	0.417	0.154	0.093
The third generation cephalosporin (J01DD)	0.024	0.803	0.004	0.961	0.000	0.996	0.132	0.151
The fourth generation cephalosporin (J01DE)	−0.080	0.393	0.064	0.471	0.143	0.097	−0.049	0.596
Carbapenems (J01DH)	0.271*	0.003	0.427*	<0.01	0.463*	<0.01	0.331*	<0.01
Fluoroquinolones (J01MA)	−0.004	0.966	0.046	0.600	0.129	0.134	0.073	0.431

^a^r denotes pearson’s correlation coefficient; *statistically significant association (*p* < 0.05)

### Correlation between carbapenem consumption intensity and CREC

**Fig. 2:**
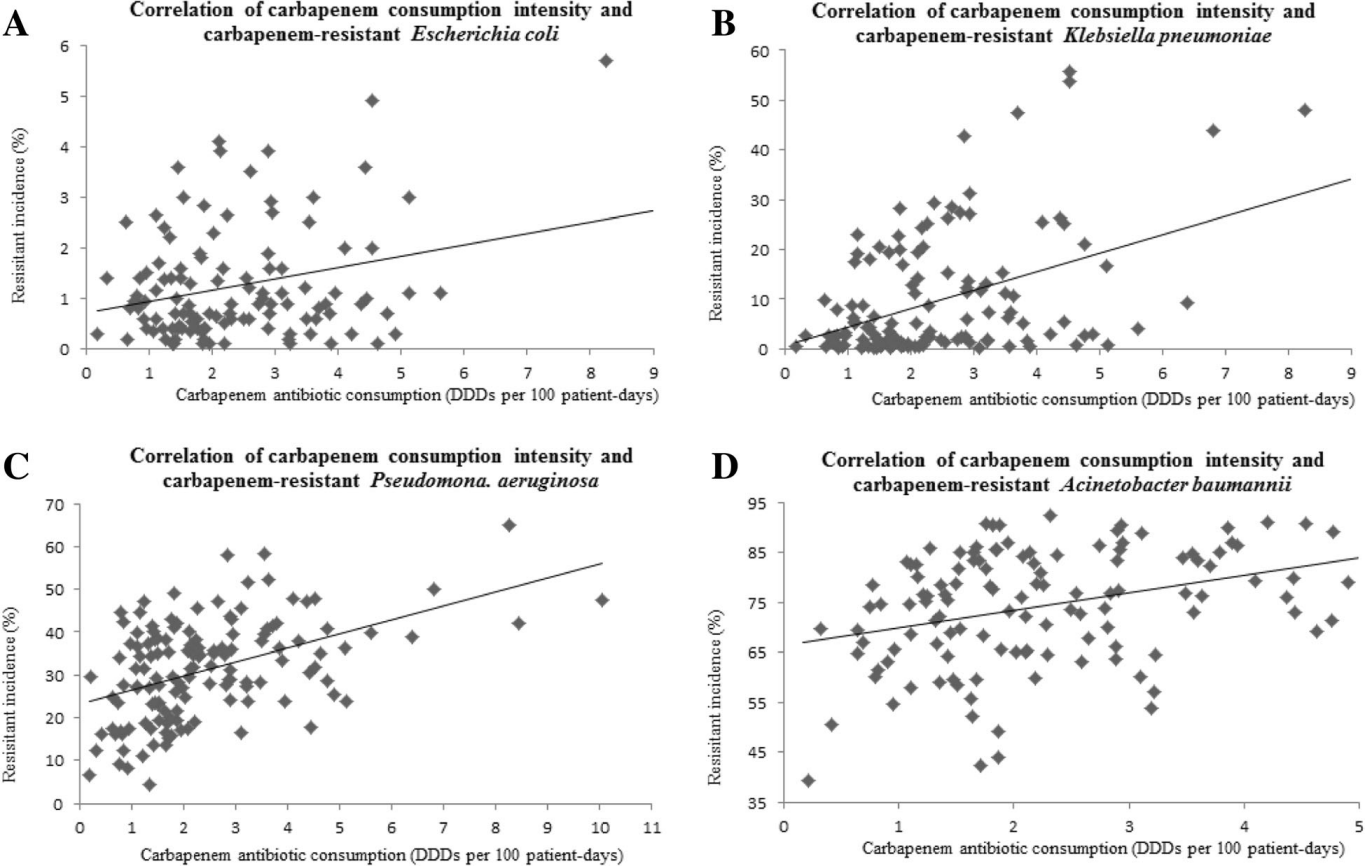
Correlation of carbapenem consumption intensity and carbapenem-resistant (**a**) *E. coli*; (**b**) *K. pneumoniae;* (**c**) *P. aeruginosa;* (**d**) *A.baumannii*

A total of 115 hospitals were studied to determine the relationship between carbapenem consumption intensity and CREC. A total of 132,974 strains of *E. coli* were isolated, 1510 strains of which were CREC. The percentage of CREC isolates in each hospital was 0.1–5.7% (median, 0.9%), and was significantly positively correlated with carbapenem antibiotic consumption (*r* = 0.271, *p* < 0.01), as demonstrated in Fig. 2a.

### Correlation between carbapenem consumption intensity and CRKP

A total of 130 hospitals were studied to determine the relationship between carbapenem consumption intensity and CRKP. A total of 97,910 strains of *K. pneumoniae* were isolated, 11,610 of which were CRKP. The percentage of CRKP isolates in each hospital was 0.1–55.6% (median, 3.38%), and was significantly positively correlated with carbapenem antibiotic consumption (*r* = 0.427, *p* < 0.01), as exhibited in Fig. 2b.

### Correlation between carbapenem consumption intensity and CRPA

A total of 136 hospitals were studied to determine the relationship between carbapenem consumption intensity and CRPA. A total of 102,739 strains of *P. aeruginosa* were isolated, 35,370 of which were CRPA. The percentage of CRPA isolates in each hospital was 4.3–65% (median, 31.60%), and was significantly positively correlated with carbapenem antibiotic consumption (*r* = 0.463, *p* < 0.01), as depicted in Fig. 2c.

### Correlation between carbapenem consumption intensity and CRAB

A total of 120 hospitals were studied to determine the relationship between carbapenem consumption intensity and CRAB. A total of 85,692 strains of *A. baumannii* were isolated, 66,047 of which were CRAB. The percentage of CRAB isolates in each hospital was 39.2–92.55% (median, 75.92%), and was significantly positively correlated with carbapenem antibiotic consumption (*r* = 0.331, *p* < 0.01), as shown in Fig. 2d.

## Discussion

This research is the first domestic study to determine the correlations between major carbapenem-resistant Gram-negative bacteria and antibiotic consumption intensity on the basis of the national surveillance data reported by 153 voluntarily participating hospitals. During the study period, cephalosporins were found to be the most commonly used antibiotics, followed by quinolones, penicillins, and carbapenems. The percentage of carbapenem-resistant Gram-negative bacteria from each hospital varied, and correlations between carbapenem consumption intensity and rate of carbapenem-resistant *E. coli, K. pneumoniae, P. aeruginosa*, and *A. baumannii* were noted.

In our study, cephalosporins were the most common antibiotic class prescribed. This finding is consistent with previous studies conducted in China [13, 14]. Unlike doctors in our country, physicians in Europe and the United States appear to prefer penicillins. This contradiction between countries may be explained by the fact that a skin test for penicillin is required prior to the administration of the antibiotic in China. Considering the time required to conduct the test, Chinese physicians prefer to prescribe cephalosporins and quinolones instead. The clinical application of carbapenem, as a specially used antibiotic, is subject to more restrictions during its prescription.

The correlation between increased carbapenem use and increased CREC found in this study is similar to that in a previous study [15]. However, in a large survey conducted in the United States, fluoroquinolone consumption was significantly correlated with the carbapenem resistance of *E. coli* [16]. Some resistant genes could spread across strains through plasmids and integrons, leading to the emergence and widespread transmission of multidrug-resistant bacteria. A multicenter study conducted in Italy detected a significantly positive correlation between the carbapenem resistance of *E. coli* and third-generation cephalosporin and penicillin consumption [17]. The use of penicillin and third-generation cephalosporins has led to the appearance of extended-spectrum beta-lactamase (ESBL), the prevalence of which could lead to increased consumption of carbapenem antibiotics and, in turn, higher proportions of CREC.

Increased use of meropenem has been associated with the rate of CRKP [15, 18]. Our study found a positive correlation between carbapenem consumption and increases in proportion of CRKP. Increased carbapenem usage prompts the production of carbapenemases, such as *K. pneumoniae* carbapenemases (KPC) and New Delhi metallo-β-lactamase-1, which could increase the proportion of CRKP [19].

Restricting carbapenems even for a short duration has been suggested to effectively manage the problem of CRPA [20]. Our study indicated that carbapenem consumption is correlated with the rate of CRPA, similar to the conclusion of a previous study in China [14]. Such a correlation may be explained by the combination of chromosomal AmpC production and porin change. AmpC enzyme overproduction, together with reduced outer membrane porin permeability and/or efflux pump overexpression, contributes to high-level carbapenem resistance [19]. Moreover, *P. aeruginosa* harbors ESBLs, including other antibiotic-resistant enzymes, such as KPC, Verona integrin-encoded metallo-β-lactamase, and imipenem metallo-훽-lactamases (IMP). The combination of these enzymes could lead to a higher rate of CRPA isolates. Research in China has revealed that quinolone consumption, besides carbapenem consumption, is substantially correlated with CRPA [14]. Another study has suggested that the CRPA rate is correlated with carbapenem plus fluoroquinolone use [21]. Quinolones and carbapenems are substrates of the same efflux pump, so the overuse of fluoroquinolones may induce the high expression of the efflux pump and lead to CRPA production [22]. Control of the usage of fluoroquinolones and carbapenems can slow down the production of multidrug-resistant *P. aeruginosa*.

A correlation between carbapenem antibiotic use and the prevalence of CRAB has been described in many previous studies [23–25]. Prior use of carbapenems is a risk factor for multidrug-resistant, extensively drug-resistant even pan drug-resistant *A. baumannii* infection [26]. This phenomenon may be attributed to selective pressure from carbapenem exposure. Excessive carbapenem use encourages the emergence of carbapenemase-producing *A. baumannii* strains, such as IMP and oxacilinase serine β-lactamases. A previous study conducted in a medical center of southern Taiwan demonstrated that carbapenem and fluoroquinolone consumption is positively correlated with increased rate of imipenem-resistant *A. baumannii.* In this particular study, fluoroquinolones were the most commonly used antibiotics [27]. The expanded use of fluoroquinolones may up-regulate the efflux pump of AdeABC. Up-regulation of efflux transcripts has been associated with a multidrug-resistant phenotype, possibly leading to CRAB production [28].

The root causes of the rapid emergence and dissemination of drug-resistant bacteria in hospitals are multifactorial, including high selective pressure resulting from improper and widespread antibiotic usage and cross-transmission from patient to patient owing to inappropriate infection control measures or interhospital transfer of resistance [29–33]. Increasing resistance may drive the increased consumption of several so-called “last-line” antibiotics and further explain why carbapenem-resistant Gram-negative bacteria are correlated with carbapenem consumption.

Despite its important findings, this study presents some limitations. First, the design of the study is retrospective, and the effects of potential confounders, such as change in the length of hospital stay, infection control practices, and hospital scale, could not be evaluated. Second, we only considered consecutive cultures, including those isolated within 48 h after hospital admission. Consequently, community-acquired isolates were not excluded from our analysis. Finally, we researched the relationship between antibiotic usage and carbapenem resistance of four common Gram-negative bacteria in the same year but did not consider the lag of the change in bacterial resistance rate. Notwithstanding these limitations, we believe that the major strength of our study is its size. We believe that, thus far, this study is the most comprehensive investigation on the topic reported domestically.

## Conclusions

In conclusion, this study highlights the positive correlation between carbapenem consumption and the rate of carbapenem resistance of four major Gram-negative bacteria. We believe that this study is a useful tool for directing antimicrobial stewardship policies. Our results also make a strong case for rationalizing the use of antibiotics to delay the occurrence of bacterial resistance. Further research on this topic may be considered in future work.

## Additional file

Additional file 1: Details of participating hospitals. (PDF 120 kb)

## Abbreviations

*A.baumannii*: *Acinetobacter baumannii*
AMR: Antimicrobial resistance
ATC: Anatomic Therapeutic Chemical
CRAB: Carbapenem-resistant *A. baumannii*
CRE: Carbapenem-resistant Enterobacteriaceae
CREC: Carbapenem-resistant *E. coli*
CRKP: Carbapenem-resistant *K. pneumoniae*
CRPA: Carbapenem-resistant *P. aeruginosa*
DDDs: Defined daily dosages
*E. coli*: *Escherichia coli*
ESBLs: Extended-spectrum beta-lactamase
IMP: Imipenem metallo-훽-lactamases
*K. pneumoniae*: *Klebsiella pneumoniae*
KPC: *K. pneumoniae* carbapenemases
*P. aeruginosa*: *Pseudomonas aeruginosa*
