# Supplementary material for: Association between antibiotic consumption and the rate of carbapenem-resistant Gram-negative bacteria from China based on 153 tertiary hospitals data in 2014
Source: Antimicrob Resist Infect Control. 2018 Nov 19;7:137. doi: 10.1186/s13756-018-0430-1 (PMC6245771; doi:10.1186/s13756-018-0430-1)
Supplement: Supplementary file 1 — Details of participating hospitals. (PDF 120 kb) [file 13756_2018_430_MOESM1_ESM.pdf]

Details of participating hospitals

Hospital number	Area	Open beds	Annual discharge(thousands people)
1	Central	3000	6
2	Central	3000	7.5
3	Central	3900	17
4	Central	3540	13.7
5	Central	1400	5.9
6	Central	4000	8
7	Central	4000	10
8	Central	5000	22.7
9	Central	2200	7
10	Central	1850	5
11	Central	2356	13.5
12	Central	1800	UN
13	Central	3500	18
14	Central	2100	7.13
15	Central	2300	7.2
16	Central	3400	7.6
17	Central	2442	9
18	Central	8475	41
19	Central	3051	10.92
20	Central	3500	14.8
21	Central	1874	UN
22	Central	3195	13
23	East	5450	19.35
24	East	4825	24.6
25	East	2464	11.3
26	East	2398	8
27	East	2800	9.4
28	East	2335	10
29	East	2631	8
30	East	2000	5.5
31	East	3300	19.2
32	East	3685	6.5
33	East	1705	3.5
34	East	2475	10
35	East	2900	14.3
36	East	1226	4
37	East	3000	10.08
38	East	2514	11
39	East	1000	3
40	East	3500	14

41	East	2813	5
42	East	2000	7
43	East	2100	10
44	East	1950	10.1
45	East	2458	11.6
46	East	3000	14.4
47	East	1903	6
48	East	6000	20
49	East	2500	13.1
50	East	2400	7.5
51	East	2685	9.43
52	North	1574	8.5
53	North	1720	8.2
54	North	1006	UN
55	North	3000	4.5
56	North	2000	9.5
57	North	1247	2
58	North	2402	7.12
59	North	2000	6
60	North	1670	6
61	North	1852	UN
62	North	2816	15
63	North	1863	10.9
64	North	2000	15
65	North	1300	4
66	North	1000	3
67	North	3092	10
68	North	3000	10
69	North	2000	5.4
70	North	1800	4
71	North	1500	5
72	North	1500	UN
73	North	1900	8.9
74	North	803	1.8
75	North	1159	5
76	North	1500	5
77	North	1800	6.45
78	North	2468	5.5
79	North	743	1
80	North	1610	5
81	Northeast	1208	3.4
82	Northeast	2350	8.41
83	Northeast	3700	10

84	Northeast	2000	7.47
85	Northeast	1450	2.2
86	Northeast	1216	6
87	Northeast	2005	15
88	Northeast	6000	20
89	Northeast	2800	UN
90	Northeast	6496	26.6
91	Northeast	1804	6.97
92	Northeast	2950	11.5
93	Northeast	2600	9.6
94	Northeast	3257	26.7
95	Northeast	1698	UN
96	Northeast	1300	4
97	Northeast	6500	23.6
98	Northwest	3200	14.03
99	Northwest	2245	4
100	Northwest	3500	11.01
101	Northwest	2686	7.5
102	Northwest	2400	9.05
103	Northwest	1200	UN
104	Northwest	3684	12.48
105	Northwest	2000	6.1
106	Northwest	2600	5.78
107	Northwest	1600	6.2
108	Northwest	3000	11.6
109	Northwest	2000	6.48
110	Northwest	1725	9
111	Northwest	2541	11.09
112	Northwest	2700	15
113	Northwest	3000	14
114	Southern	1206	4
115	Southern	2852	11.06
116	Southern	1371	6.28
117	Southern	837	2
118	Southern	2750	10
119	Southern	2117	7.95
120	Southern	2490	8.75
121	Southern	720	1.2
122	Southern	1000	4.5
123	Southern	1057	3.5
124	Southern	1950	4.6
125	Southern	1800	4.5
126	Southern	3000	8.5

127	Southern	1462	4.4
128	Southern	2000	8
129	Southern	3095	12.4
130	Southern	2225	10.43
131	Southern	1300	UN
132	Southern	1800	6.4
133	Southern	2500	10.1
134	Southern	2179	10
135	Southern	2818	11.34
136	Southern	1800	5.4
137	Southern	2850	10.61
138	Southern	3200	13.1
139	Southern	1500	5
140	Southwest	2860	12
141	Southwest	2900	13.2
142	Southwest	3000	10
143	Southwest	3000	9.2
144	Southwest	2100	UN
145	Southwest	1500	4.58
146	Southwest	4000	15
147	Southwest	4200	12
148	Southwest	1500	9
149	Southwest	4300	24.32
150	Southwest	4123	UN
151	Southwest	1000	2.2
152	Southwest	1380	4.5
153	Southwest	3200	14

UN：unknown
